# Effects of Aflatoxin B_1_ and Fumonisin B_1_ on the Viability and Induction of Apoptosis in Rat Primary Hepatocytes

**DOI:** 10.3390/ijms11041944

**Published:** 2010-04-26

**Authors:** Deise H. B. Ribeiro, Fabiane L. Ferreira, Valéria N. da Silva, Simone Aquino, Benedito Corrêa

**Affiliations:** 1 Centro de P&D de Proteção Ambiental, Instituto Biológico, Av. Conselheiro Rodrigues Alves n. 1252 – Vila Mariana. São Paulo, SP. 04014-002, Brazil; 2 Departamento de Microbiologia, Instituto de Ciências Biomédicas – Universidade de São Paulo, Av. Prof. Lineu Prestes n. 1374 – Cidade Universitária. São Paulo, SP. 05508-900, Brazil

**Keywords:** aflatoxin B_1_, apoptosis, fumonisin B_1_, primary hepatocytes

## Abstract

The present study evaluated the effect of aflatoxin B_1_ (AFB_1_) and fumonisin B_1_ (FB_1_) either alone, or in association, on rat primary hepatocyte cultures. Cell viability was assessed by flow cytometry after propidium iodine intercalation. DNA fragmentation and apoptosis were assessed by agarose gel electrophoresis and acridine orange and ethidium bromide staining. At the concentrations of AFB_1_ and FB_1_ used, the toxins did not decrease cell viability, but did induce apoptosis in a concentration and time-dependent manner.

## 1. Introduction

Aflatoxins are biologically active metabolites produced by the *Aspergillus* strains *A. flavus*, *A. parasiticus*, *A. nomius*, and *A. pseudotamarii* [1]. The biological response to aflatoxin B_1_ (AFB_1_) in terms of genotoxicity and cytotoxicity depends on the metabolic formation of AFB_1_-8,9-epoxide [2], which can covalently bind to nucleic acids or proteins, provoking cell membrane damage, necrosis and mutagenesis in the affected cells [3].

Fumonisins are secondary metabolites produced mainly by *Fusarium verticillioides* and *F. proliferatum* [4,5]. Their mechanism of action is still not completely understood; however, these toxins show an extraordinary structural similarity to sphingoid bases, sphinganine and sphingosine, and as such selectively inhibit ceramide synthetase, a key enzyme in the biosynthetic pathway of sphingolipids [6], which leads to an increase in the levels of sphinganine and occasionally to sphingosine in the cell [7].

Apoptosis is a specialized process of cell death that is part of the normal development of organs and tissue maintenance, but may also occur as a response to various environmental stimuli, indicating toxicity. Since apoptosis can play a critical role in the development of cancer, the ability of toxins to induce apoptosis appears to be related to their toxicological effects [8].

One of the initial events that occurs in the target organs exposed to fumonisin is apoptosis, which might be a consequence of the inhibition of ceramide synthetase and alterations in sphingolipid metabolism [9]. Another mechanism of apoptosis induction probably involves the inhibition of protein kinase C [10], a key hallmark of apoptosis signaling [11] and destructuring of the endothelial barrier [12].

The regulation of sphingolipid biosynthesis is essential for the cell, since its products affect the cellular behavior; ceramide and sphingosine are cytotoxic, inhibit growth and are able to induce early apoptosis. Sphingosine can be phosphorylated by sphingosine kinase to form sphingosine-1-phosphate, a potent mitogen that antagonizes the apoptotic effect mediated by ceramide. A model has been proposed in which the balance between the intracellular levels of ceramide and sphingosine-1-phosphate could determine whether a cell survives or dies [13].

The co-occurrence of fumonisin B_1_ (FB1) and AFB_1_ has been reported by several researchers as a worldwide problem [14]. Although the clinical, biochemical, hematological and mycotoxicological parameters of both toxins acting alone have been well studied [15], at present, little is known about the interaction of AFB_1_ and FB_1_ regarding their toxic and carcinogenic properties and the interactive effects of these toxins *in vivo.*

The present study describes the isolated and combined effects of AFB_1_ and FB_1_ on rat primary hepatocyte cultures submitted to different treatments, based on the analysis of cell viability, apoptosis induction detected by DNA fragmentation and observation of morphological alterations.

## 2. Experimental Section

### 2.1. Animals and Cell Isolation

Forty Male Wistar rats (45 days-old) were obtained from the Animal House of the Veterinary School at the University of São Paulo (USP). The rats were maintained in boxes and fed with Nuvital^®^ chow and water *ad libitum* until euthanasia in a CO_2_ chamber.

Primary hepatocytes were obtained by the collagenase disaggregation technique [16]. The liver was excised, cleaned of undesired tissues, cut into small pieces (1 mm^3^) and washed with Phosphate Buffered Saline (PBS) to remove any red blood cells. Opti-MEM^®^ cell culture medium containing 10% fetal bovine serum, 200 U/mL collagenase, 100 U/mL penicillin and 100 μg/mL streptomycin was then added to the tissue and the processed material was incubated at 37 °C for 4 h.

### 2.2. Cell Culture

After cell disaggregation, the collagenase was removed by centrifugation. The cells were resuspended in Opti-MEM^®^ medium containing 10% fetal bovine serum, their viability was checked by trypan blue exclusion and they were seeded at a high concentration (5,000–6,000 viable cells) onto 24-well plates precoated with collagen. The plates were incubated at 37 °C in a 5% CO_2_ atmosphere. The cells were plated 24 h before initiating the experiment. They were detached with 0.25% trypsin and 0.03% versene prior to performing flow cytometry analysis and the observation of morphological alterations.

### 2.3. Toxins

The AFB_1_ used in the experiment was produced at Laboratory of Mycotoxins of the Biomedical Science Institute (USP), using toxigenic strains of *Aspergillus flavus* IMI 190. A small fragment of an *A. flavus* colony was inoculated in the center of a Petri dish with coconut agar. Incubation was conducted at 25 °C for 10 days, after which the cultures were assayed for aflatoxins, as described by Lin and Dianese (1976) [17]. The coconut agar cultures were extracted with chloroform (30 mL chloroform per 10 g of culture) by shaking for 30 min. The content was filtered through a Whatman #1 filter paper and evaporated to dryness. Purification was achieved with hexane and partitioned with chloroform, resulting in a solution of 96% AFB_1_ and 4% AFB_2_. Quantification was achieved by densitometry, as described by Oliveira *et al*. (2002) [18]. Given the toxicity of chloroform, the solution was placed in flasks, kept in a water bath at 60 °C until complete evaporation of the diluent and subsequently resuspended in dimethyl sulfoxide (DMSO). The concentrations of the standard were established according to the Manual of Official Methods of the Association of Official Analytical Chemistry [19].

The purified FB_1_ (95%) used in the inoculation of the cell cultures was acquired from The Programme on Mycotoxins and Experimental Carcinogenesis (PROMEC Unit) of the South African Medical Research Council (MRC), South Africa, and was diluted in PBS.

### 2.4. Treatments

Primary hepatocytes were incubated in culture medium containing one of the following concentrations: T1, 9 nM AFB_1_; T2, 50 μM FB_1_; T3, 9 nM AFB_1_ + 10 μM FB_1_; T4, 9 nM AFB_1_ + 50 μM FB_1_; T5, 9 nM AFB_1_ + 100 μM FB_1_; and T6, 9 nM AFB_1_ + 250 μM FB_1_. The control group was incubated with 0.25% DMSO alone. All experiments were performed in triplicate and the cells were assayed after 1 to 4 h.

### 2.5. Flow Cytometry Analysis

Primary hepatocytes treated as described in Section 2.4; following trypsinization, 10^6^ cells were resuspended in 5 mL D-PBS. Propidium iodide (10 ng/mL) was added and incubated for 10 min at 37 °C, washed with PBS and resuspended in 0.8 mL PBS. The treated cells were subjected to flow cytometric analysis (20000 events) using a FACScan (Becton Dickinson, San Jose, CA) by observing the propidium iodide (564–606 nm). The distribution of cells and the percentage of dye-labeled cells were determined using the CellQuest software (Becton Dickinson).

### 2.6. Detection of DNA Fragmentation by Agarose Gel Electrophoresis

After removal of the culture medium, the cells were washed with PBS and 0.5 mL lysis buffer (100 mM hydroxymethyl aminomethane hydrochloride (Tris-HCl), pH 8.5, 5 mM ethylenediamine tetraacetic acid (EDTA), 0.2% sodium dodecyl sulfate (SDS), 200 mM NaCl, 100 μg/mL proteinase K) was added to each well. The plates were incubated overnight at 37 °C. After shaking for 30 min at room temperature, 0.5 mL of isopropanol was added to each well and the plates were again shaken until complete DNA precipitation. The DNA was transferred to a microtube and washed with 100 μL of 70% ethanol. Next, the ethanol was removed and the microtubes were left open to dry for approximately 15 min. The DNA was resuspended in 25 μL TE buffer (10:1 Tris-HCL:EDTA) and incubated overnight at 55 °C to optimize dissolution.

**Figure 1.**
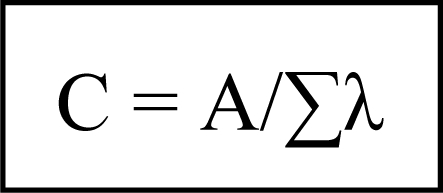
Equation used to quantify the DNA.

DNA purity was checked by reading at an optical density (OD) of 260 and 280 nm using a spectrophotometer. The OD260/OD280 ratio was calculated and a value of 1.8 indicated pure DNA with absence of contaminants, such as proteins, RNA and phenols. DNA quantification was determined by diluting 10 μL of DNA and 990 μL of TE buffer and reading at OD 260 (Figure 1).

Samples of exactly 20 μg of DNA were submitted to 1.2% (w/v) agarose gel electrophoresis in 0.5 × TAE buffer (Tris-Acetate-EDTA) containing 0.5 μg/mL ethidium bromide at 100 mV for 60 min.

### 2.7. Observation of Morphological Alterations

One and 4 h after toxin inoculation, 25 μL of the cell suspension (10^3^ cells) was incubated with 1 μL of the following solution: 1 part acridine orange (100 μg/mL)/1 part ethidium bromide (100 μg/mL). The slides were observed under a fluorescence microscope, almost 300 cells were counted and the images were analyzed using Image Pro Plus software [20].

### 2.8. Statistical Analysis

The results are reported as mean ± standard error of the mean. Differences between treatments over time were analyzed statistically by analysis of variance using a general linear model procedure and considering two factors: treatment (6 levels) and time (4 levels). A mean profile graph was constructed to illustrate the results [21]. A significance level of 0.05 was adopted. Statistical analysis was performed using the SPSS (version 11.0) and SAS (version 8.01) programs.

## 3. Results and Discussion

**Figure 2.**
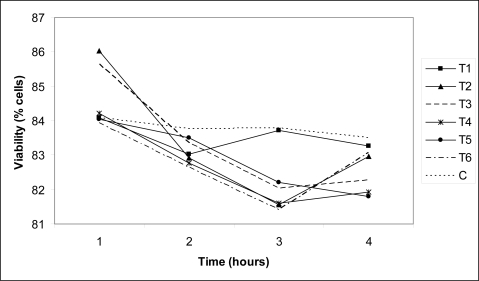
Percentage of viable cells after one, two, three and four hours of incubation with: T1, 9 nM AFB_1_; T2, 50 μM FB_1_; T3, 9 nM AFB_1_ + 10 μM FB_1_; T4, 9 nM AFB_1_ + 50 μM FB_1_; T5, 9 nM AFB_1_ + 100 μM FB_1_; T6, 9 nM AFB_1_ + 250 μM FB_1_; and C, control. These data were obtained using general linear model procedures

Analysis of the mean profile of the percentage of cell viability (Figure 2) revealed no differences between the different concentrations of FB_1_ and AFB_1_ (p = 0.8320). Cell viability decreased after one hour of incubation in all treatments compared to controls (p = 0.004). Statistically, a time-dependent effect (p = 0.0056) was observed; when comparing the viability data at two, three and four hours, no significant difference occurred between them in any of the treatments (p = 0.4274). In the present experiment, although a decrease in viability occurred after one hour, it was not relevant when considering that viability ranged from 82% to 84%. After four hours of exposure, the hepatocytes incubated with 9 nM AFB_1_ presented 83% of viable cells. Similar findings have been reported by Shen *et al*. (1996) (22), who demonstrated that more than 90% of cells treated with 10 nM AFB_1_ continued to be viable after four hours of incubation.

A clear concentration-dependent increase in the cytotoxicity of bovine primary hepatocytes was observed by Kuilman *et al*. [23] using AFB_1_ doses and exposure times that were much higher than those used in the present study. The authors reported less than 40% viability for cells treated with 16 μM AFB_1_ after 24 hours. When studying rat hepatocytes incubated with AFB_1_, Metcalfe and Neal [24] also reported signs of cytotoxicity that were proportional to the increase in the doses and exposure times studied.

The effects observed for the isolated administration of FB_1_ are in agreement with those reported by several researchers. No evident alteration in cell viability irrespective of treatment and exposure time to FB_1_ has been reported in human fibroblasts [25], endothelial cells isolated from pig pulmonary artery (12) or in a human erythroleukemia cell line (K562) [26].

In this experiment, the simultaneous administration of different concentrations of FB_1_ and AFB_1_ did not significantly alter cell viability compared to cells incubated with either toxin alone. Despite the toxin and concentration used, cell viability decreased after one hour of incubation, suggesting that the cellular viability may decrease in a time-dependent manner.

In 1995, Wu *et al*. [27] demonstrated that FB_1_ was not cytotoxic to cardiomyocytes, skeletal myocytes, hepatocytes or macrophages, but its effects were clearly visible in splenocytes and chondrocytes of primary chicken cell cultures.

In contrast, some researchers have reported dose- and/or time-dependent effects of FB_1_ on some cell lines. While studying rabbit kidney epithelial cells (RK13), Rumora *et al*. [28] observed impaired cell viability after 24 hours of exposure. The same effects were reported for murine macrophages (RAW264.7) [29], pig kidney epithelial cells (LLC-PK_1_) [30], the C6 cell line [31] and for human colon carcinoma cells (HT29) [32].

The effect of FB_1_ on the number of viable cells becomes clearer when taking into account the effects of this toxin on the cell cycle. In this respect, Bouhet *et al.* [33] analyzed the effects of FB_1_ on the cycle of intestinal (IPEC-1) and renal (LLC-PK1) epithelial cells and observed that the inhibition of G0 to G1 cell cycle progression was concomitant with the effects on cell proliferation. The apoptosis induced by FB_1_ or cell cycle arrest was reported in CV-1 cells (African green monkey kidney fibroblasts) [34] and in rat hepatocytes [35], suggesting cell cycle-dependent cytotoxicity as a potential mechanism underlying the hepatocarcinogenicity of FB_1_. Other studies have indicated that FB_1_ may inhibit cell cycle progression in different phases; for example, cell cycle arrest in the G2/M phase was observed in rat brain glioma cells (C6) [31] and in esophageal carcinoma cells (WHCO3) [36].

**Figure 3.**
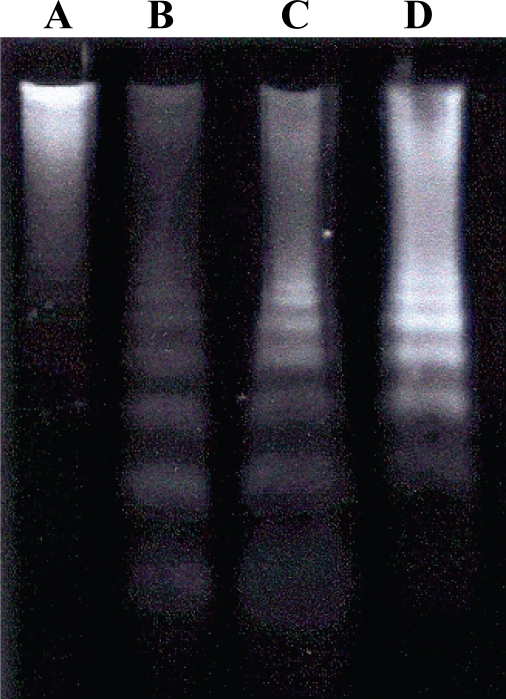
DNA ladder formation in primary hepatocyte after three hours of exposure. **A:** Control (0.26% DMSO); **B:** 9 nM AFB_1_; **C:** 50μM FB_1_; **D:** 9 nM AFB_1_ + 10 μM FB_1_.

Nucleosomal DNA fragmentation was observed in cells treated with FB_1_, AFB_1_ or a combination of these toxins. Fragmentation was more visible in cells treated with both toxins than with either toxin alone (line D of Figure 3). It was possible to determine which sample presented the most pronounced effect since the expression of the effect was determined using the same amount of DNA (20 μg) for each experiment.

The classical biochemical marker of apoptosis is DNA laddering into oligosomes mediated by the endonucleolytic activity of caspase-3. Agarose gel electrophoresis of nuclear DNA extracted from apoptotic cells shows a typical ladder banding pattern, since in this type of cell death, DNA fragmentation generally results in segments of approximately 180 to 200 bp and their multiples. In the case of necrosis, DNA fragmentation is random and the electrophoretic pattern is revealed as a screen [37].

The formation of a ladder pattern after incubation with FB_1_ has also been observed in African green monkey kidney cells (CV-1) [34] and in human neonatal keratinocytes (NHKc) [38]. These studies suggested that these alterations are related to the accumulation of sphinganine.

According to Seefelder *et al*. [9], the characteristic formation of a DNA ladder and the condensation of chromatin in human immortalized proximal tubule cells (IHKE) is due to an increase in caspase-3 activity.

In contrast, DNA fragmentation was not observed in immortalized rabbit epithelial cells (RK13); however, the cells showed alterations in cell morphology compatible with the induction of apoptosis [28]. An absence of the nuclear effects of apoptosis seems to occur in certain cell lines, including WHCO3 esophageal cancer cells [36] and endothelial cells isolated from pig pulmonary artery, both incubated with FB_1_ [12].

Analysis of the results showed that the concentrations used do not significantly diminish cell viability during the treatment; it seems that FB_1_ did not delay or accelerate the decrease in cell viability.

The data as presented indicate that 9 nM AFB_1_ caused cell death, including apoptosis in primary hepatocytes. Apoptosis induction also seems to be a concentration and time-dependent effect when FB_1_ and AFB_1_ are used in association. These data demonstrated that the enhanced apoptosis was due to the increased levels of FB_1_.

Viable cells and cells in early and late apoptosis can be differentiated using acridine orange/ethidium bromide. Apoptosis induction can be observed based on morphological alterations and by the staining properties of the cells; *i.e.,* differential uptake of the dyes is an important component in this differentiation.

Acridine orange is a vital dye that stains both live and dead cells, ethidium bromide only stains cells that have lost membrane integrity, while live and heathy cells appear uniformly stained. Early apoptotic cells stain green and contain bright dots in the nuclei. Late apoptotic cells also incorporate ethidium bromide and show condensed and often fragmented nuclei. Necrotic cells also stain in orange, but present nuclear morphology resembling that of viable cells.

Morphologically, the cells were classified as either live or apoptotic. Apoptotic cells are marked by cellular shrinking, condensation and margination of the chromatin and ruffling of the plasma membrane, called budding. Eventually the cell becomes divided into apoptotic bodies that consist of cell organelles and/or nuclear material surrounded by an intact plasma membrane. According to Van Cruchten and Van den Broeck, hepatocyte apoptosis is characterized by peculiar morphological features, including decreased cell volume, ruffled cell membranes, condensed chromatin beneath the nuclear envelope and, eventually, cell segregation with the formation of numerous vesicles containing intact organelles [39].

Characteristic morphological effects were observed in cells exposed to the toxins, thus confirming the results obtained by DNA fragmentation analysis.

**Figure 4.**
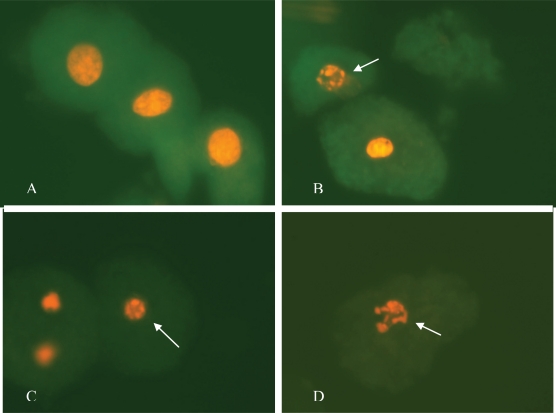
Photomicrography of the primary rat hepatocytes submitted to the following treatments: **A:** Control (0.26% DMSO); **B:** 9nM AFB_1_ + 100μM FB_1_; **C:** 9nM AFB_1_ + 250μM FB_1_; **D:** 9nM AFB_1_, stained with acridine orange and ethidium bromide (original magnification x600). Arrows indicate apoptotic nuclei.

Although few cells showed evident apoptotic features after being treated with 9 nM of AFB_1_, at the highest concentration of toxin association (FB_1_ = 250 μM + AFB_1_ = 9nM), the signs included nuclear chromatin condensation and increased staining intensity and disintegration of the nuclear envelope and membrane-packaged DNA. At this point, the number of cells in late apoptosis was three-fold higher than the previous concentration (FB_1_ = 100 μM + AFB_1_ = 9nM), as shown in Figure 4.

At the other concentrations of FB_1_ alone and in association with AFB_1_, cellular dehydration was evident, with modifications in shape and size, as well as chromatin condensation and hyperchromasia.

The data suggested that the promotion of an apoptotic effect by FB_1_ increased in a concentration and time-dependent manner and this effect was further highlighted when this toxin was administered in association with AFB_1._ *In vivo* and *in vitro* experiments suggest that FB_1_ behaves in a manner similar to most cancer promoters by inducing differential growth in initiated cells [40].

Others authors reported similar results when conducting *in vitro* studies, verifying that coexposure to fumonisins and AFB_1_ produced greater liver toxicity compared to their administration alone, inducing apoptosis and mitotic hepatocytes. Inversion of the typical Sa:So ratio occurred in rats [41]; therefore, the mixture of fumonisins and AFB_1_ induced toxic responses that could not be considered the sum of the effects caused individually by these mycotoxins [42].

In the present study, the morphological alterations observed, the ladder-like electrophoretic pattern and chromatin condensation accompanied by nuclear fragmentation, indicate that the effects on the cells was caused by chromatin breakage, thus characterizing cell death by apoptosis. These results are in agreement with those obtained studying nonimmortalized human fibroblasts exposed to FB_1_, in which the DNA damage observed was dose-dependent [25]. Modifications corresponding to early apoptosis have also been demonstrated by Minervini *et al*. [26] in K563 human erythroleukemia cells submitted to treatment with FB_1_.

The interaction between different natural occurring toxins, such as aflatoxins and fumonisins are very complex and the present study indicates that more emphasis should placed on these interactions to aid in the elucidation of the toxicological and carcinogenic effects of these toxins.
